# The Effects of Fibrotic Cell Type and Its Density on Atrial Fibrillation Dynamics: An In Silico Study

**DOI:** 10.3390/cells10102769

**Published:** 2021-10-15

**Authors:** Laura C. Palacio, Juan P. Ugarte, Javier Saiz, Catalina Tobón

**Affiliations:** 1 Materiales Nanoestructurados y Biomodelación (MATBIOM), Universidad de Medellín, Medellín 050032, Colombia; lcpt91@gmail.com; 2 Grupo de Investigación en Modelamiento y Simulación Computacional (GIMSC), Universidad de San Buenaventura, Medellín 050010, Colombia; juan.ugarte@usbmed.edu.co; 3 Centro de Investigación e Innovación en Bioingeniería (CI^2^B), Universitat Politècnica de València, 46022 Valencia, Spain; jsaiz@ci2b.upv.es

**Keywords:** diffuse fibrosis, atrial fibrillation, 3D models, electrograms

## Abstract

Remodeling in atrial fibrillation (AF) underlines the electrical and structural changes in the atria, where fibrosis is a hallmark of arrhythmogenic structural alterations. Fibrosis is an important feature of the AF substrate and can lead to abnormal conduction and, consequently, mechanical dysfunction. The fibrotic process comprises the presence of fibrotic cells, including fibroblasts, myofibroblasts and fibrocytes, which play an important role during fibrillatory dynamics. This work assesses the effect of the diffuse fibrosis density and the intermingled presence of the three types of fibrotic cells on the dynamics of persistent AF. For this purpose, the three fibrotic cells were electrically coupled to cardiomyocytes in a 3D realistic model of human atria. Low (6.25%) and high (25%) fibrosis densities were implemented in the left atrium according to a diffuse fibrosis representation. We analyze the action potential duration, conduction velocity and fibrillatory conduction patterns. Additionally, frequency analysis was performed in 50 virtual electrograms. The tested fibrosis configurations generated a significant conduction velocity reduction, where the larger effect was observed at high fibrosis density (up to 82% reduction in the fibrocytes configuration). Increasing the fibrosis density intensifies the vulnerability to multiple re-entries, zigzag propagation, and chaotic activity in the fibrillatory conduction. The most complex propagation patterns were observed at high fibrosis densities and the fibrocytes are the cells with the largest proarrhythmic effect. Left-to-right dominant frequency gradients can be observed for all fibrosis configurations, where the fibrocytes configuration at high density generates the most significant gradients (up to 4.5 Hz). These results suggest the important role of different fibrotic cell types and their density in diffuse fibrosis on the chaotic propagation patterns during persistent AF.

## 1. Introduction

Atrial fibrillation (AF) is the most common arrhythmia in clinical practice [1], with important implications on public health [2,3]. The mechanisms of initiation and maintenance of AF include ectopic foci [4], multiple re-entrant waves [5] and rotors [6]. The AF is characterized by a process of atrial remodeling, that includes electrical and structural alterations contributing to the formation of arrhythmogenic substrates [7,8]. The structural remodeling process includes fibrosis, which is attributed to an excessive deposition of extracellular matrix components, such as collagen by means of activated cardiac fibroblasts [9]. Recent studies have shown that external factors, such as air pollution, increase the cardiovascular and heart arrhythmias risk. Specifically, the exposure to particulate matter contributes to adverse remodeling and a worsening of myocardial fibrosis [10,11,12,13].

Fibrotic remodeling of atrial tissue involves processes occurring in parallel across multiple scales. The differentiation of fibrotic cells at the cellular level [14] and excessive deposition of collagen at the tissue level [15], yield the conditions for generating of complex propagation dynamics [16]. Several studies suggest that the amount of fibrosis is associated with conduction slowing and increased vulnerability to cardiac arrhythmias [16,17], and maintenance of AF [15,18]. Additionally, the fibrosis texture (compact, patchy or diffuse), plays a crucial role in determining the conduction abnormalities [19]. Compact fibrosis comprises a fibrotic area that is deprived of myocardium, although severe at first glance, is the least arrhythmogenic among the different types of fibrosis. Patchy fibrosis is characterized by areas where collagen fibers of long strands and myocardial bundles intermingle. This type of fibrosis can cause conduction delays because of zig-zag conduction between the various bundles and is vulnerable to arrhythmias [20]. Diffuse fibrosis consists of short stretches of fibrosis, and it also impairs conduction. Several studies [21,22] have found that diffuse fibrosis has a significant effect on propagation, it reduces the velocity conduction and leads to the formation of re-entrant waves. In addition, diffuse fibrosis increases the cycle length of re-entrant arrhythmias and favors the transition from tachycardia to fibrillation [19]. Although significant progress has been made in the understanding of the relationships between the distinct factors playing during the fibrosis process and the resulting arrhythmogenic substrate, crucial gaps still exist.

The presence of fibroblasts, myofibroblasts and fibrocytes has been reported in cardiac fibrosis [23,24,25,26,27]. Fibroblasts are the main cellular source for the synthesis of the extracellular matrix, they are activated in a reparative or reactive response to tissue damage [28] and their production increases under conditions of AF [29]. Fibroblasts can also phenotypically transform into myofibroblasts and participate in fibrogenesis [30]. In addition to fibroblasts population in the myocardium, other cells of hematopoietic and extracardiac origin known as fibrocytes can transform into myofibroblasts, further driving the fibrotic response [23]. The proliferation of fibroblasts and myofibroblasts has been associated with atrial conduction abnormalities and delays in the propagation of the cardiac electrical impulse [16,31,32], which represent an arrhythmogenic substrate for atrial re-entries [33]. There is experimental evidence that the action potential propagation is affected by the electrical coupling between cardiomyocytes and fibroblasts [25] or myofibroblasts [27,31,34]. Several studies have shown that myofibroblasts can result from the differentiation of cells with extracardiac origin, such as the fibrocytes [35]. Fibrocytes may be involved in atrial fibrosis during AF [23] and its presence has been reported in atrial tissue with conduction blocks [36]. The growing evidence of the relationship between the fibrosis process and the AF initiation and perpetuation, motivates further studies to improve the understanding of the specific role and interplay of fibroblasts, myofibroblasts and fibrocytes during AF [37].

The isolation of the mechanisms by which diffuse fibrosis and its components contribute to AF is a difficult task in an experimental setup. A computational model, with a realistic description of the atrial geometry and electrophysiology, representing different configurations and densities of fibrotic lesions, becomes a useful tool to explore the contribution of fibrosis and their components on AF progression and to predict them in living models and real patients. In this study, we evaluate the effect of low and high diffuse fibrosis density and the intermingled presence of the three types of fibrotic cells (fibroblast, myofibroblast, fibrocytes and their combination) on the AF propagation dynamics by means of computational simulations.

## 2. Materials and Methods

### 2.1. Atrial Myocyte under Atrial Fibrillation Remodeling

The mathematical model of the human atrial cell, developed by Courtemanche et al. [38], was implemented. To represent the cholinergic activity, the equation for the acetylcholine-dependent current was included (*I_KACh_*) [39]. The membrane potential of the atrial cardiomyocyte (*V_m_*) is calculated using the following Equation (1):

(1) $$\frac{dV_{m}}{dt}=\frac{-\left(I_{ion}+I_{st}\right)}{C_{m}},$$

where, *C_m_* is the membrane capacitance of the cardiomyocyte (100 pF), *I_ion_* is the total transmembrane ionic current, and *I_st_* is the external stimulation current. The resting membrane potential is set to −81.2 mV. To simulate the conditions of persistent AF electrical remodeling, the conductivity of different ionic channels was modified based on experimental studies [40,41,42]. The implementation of the electrical remodeling is different in both atria: the conductivity of the transient outward potassium channel (*I_to_*) is reduced by 45% and 75%, and ultra-rapid outward channel (*I_Kur_*) by 60% and 45%, in the right and left atrium, respectively. The conductivity of the delayed rectifier potassium channel (*I_Ks_*) is increased by 150% in the right atrium and by 100% in the left atrium. In both atria, conductance of the inwardly rectifying potassium channel (*I_K1_*) is increased by 100% and conductance of the current through the L-type calcium channel (*I_CaL_*) is reduced by 65%. Additionally, the conductivity of atrial myocardium was reduced by 15%, yielding a value of 0.26 S/cm [43,44].

### 2.2. Fibroblast, Myofibroblast and Fibrocyte Model

The mathematical model proposed by MacCannell et al. [45] was adopted for representing fibroblasts and it was also modified to implement myofibroblasts and fibrocytes. The membrane potential for a fibrotic cell (*V_cfi_*) is described using the following Expression (2):

(2) $$\frac{dV_{cfi}}{dt}=-\frac{I_{cfi}}{C_{cfi}},$$

where, *I_cfi_* and *C_cfi_* are the transmembrane current and the membrane capacitance of the fibrotic cell, respectively. In agreement with experimental and computational studies [45,46,47,48,49], the resting membrane potential is set to −49.6 mV for all fibrotic cells. The membrane capacitance values are set to 6.3 pF for fibroblasts and fibrocytes, and 50.4 pF for myofibroblasts. The conductivity of fibroblasts and myofibroblasts is set to 0.078 mS/cm (i.e, 30% of the cardiomyocytes conductivity). Based on studies reporting conduction blockades in the atrial tissue in the presence of fibrocytes [36,50], a low value of conductivity (i.e., 0.024 S/cm) was assigned to the fibrocytes. Table 1 summarizes the cardiomyocyte and fibrotic cells parameters configuration.

### 2.3. Diffuse Fibrosis in a 3D Model of Human Atria

**Figure 1:**
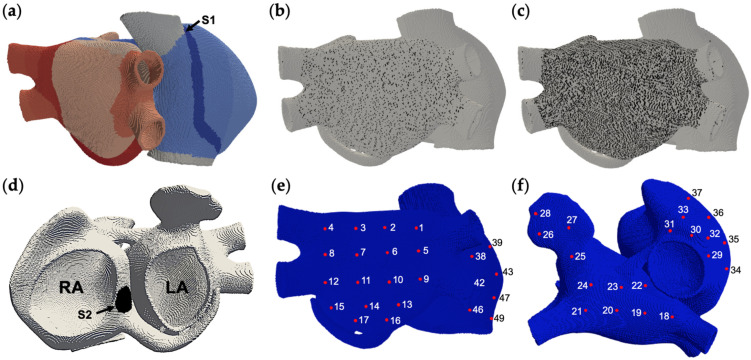
The 3D model of human atria. (**a**) Atrial regions with heterogeneity and sinoatrial node (S1) are shown. The fibrosis distribution in the left atrium is shown at densities of (**b**) 6.25% (low) and (**c**) 25% (high). (**d**) Ectopic focus location (S2), where right (RA) and left (LA) atrium are indicated. (**e**,**f**) 50 virtual electrodes locations.

A 3D model of human atria, developed in a previous work, is implemented [53,54]. The mesh is composed by 515010 hexahedral elements with uniform spatial resolution of 300 μm (Figure 1a). The model includes the main anatomical structures, realistic fiber orientation, electrophysiological heterogeneity and anisotropy, as described in [54].

Atrial fibrosis is implemented by considering cardiomyocytes coupled to fibrotic cells. Such heterocellular couplings are randomly distributed over the left atrium of the 3D model aiming to represent a diffuse fibrosis texture. Seven configurations of fibrosis were implemented according to the electrical coupling of cardiomyocytes with: (a) fibroblasts, (b) myofibroblasts, (c) fibrocytes, (d) fibroblasts and myofibroblasts, (e) fibroblasts and fibrocytes, (f) myofibroblasts and fibrocytes; and (g) fibroblasts, myofibroblasts and fibrocytes. The density of fibrosis was defined in agreement with a clinical study in patients with AF [55]. In such study, the patients were classified according to the Utah stages, which define each stage according to the percentage of fibrosis, with respect to the volume of the left atrium, quantified using magnetic resonance imaging. For each fibrosis configuration, the densities of 6.25% (low) and 25% (high) are tested, thus obtaining a total of 14 fibrosis scenarios. These fibrosis densities are obtained by assigning the heterocellular couplings to 6.25% (Figure 1b) and 25% (Figure 1c) of the total number of nodes in the left atrium (excluding pulmonary veins, junctions with the coronary sinus, Bachmann bundle and interatrial septum) for low and high density, respectively. The fibrotic cell model (fibroblast, myofibroblast or fibrocyte) is assigned to the nodes with heterocellular couplings. Fibrotic cells are arranged so that they are uniformly interspersed with cardiomyocytes. For configurations including more than one fibrotic cell, the number of nodes for each cell is homogeneous. The fibrotic cell type is randomly assigned to the nodes defined as containing heterocellular couplings until reaching the expected densities. The low and high densities correspond to the Utah stage 1 (<8.5%) and Utah stage 4 (>21%), respectively [55]. If at least one node within an element of the mesh, corresponds to a heterocellular coupling, the conductivity of the corresponding fibrotic cell is assigned to the element, as reported in previous simulation studies [56].

### 2.4. Model of Electrical Propagation

The monodomain equation representing the cardiac action potential propagation is described by the following reaction-diffusion equation [57]:

(3) $$\nabla \cdot \left(D\nabla V\right)=C\frac{\partial V}{\partial t}+I+I_{st},$$

where *C*, *V* and *I* are the membrane capacitance, potential and transmembrane current of the cardiac cell (cardiomyocyte or fibrotic cell), respectively. Additionally, *D* stands for the conductivity tensor. The Equation (3) was numerically solved using the finite element method implemented in the EMOS software [58], with a constant time step of 0.01 ms.

### 2.5. Stimulation Protocol and Simulation Setup

The S1–S2 standard stimulation protocol was implemented, where S1 simulates the sinus rhythm, and it is applied at the sinus node located in the wall of the right atrium. The S2 stimulus represents an ectopic focus, and it is applied at the interatrial septum near to the coronary sinus (Figure 1d). The S2 is a train of 5 beats at a cycle length of 110 ms. The first stimulus of S2 was applied to a proper coupling interval that generates a unidirectional block. Each stimulus consists of rectangular pulses with a duration of 2 ms and amplitude of 28 pA/pF.

The simulation time was set to 5 s. The action potential duration at 90% of the repolarization (APD_90_) was calculated in the center of the posterior wall of the left atrium. The conduction velocity was measured using two nodes located at the posterior wall of the left atrium.

### 2.6. Electrograms and Dominant Frequency

Unipolar electrograms (EGM) were calculated as extracellular potentials (*Φ_e_*) recorded by virtual electrodes located at 0.2 mm from the atria surface. The extracellular potential is obtained using the following formulation (4) [59]:

(4) $$\Phi_{e}(\vec{r})=-\frac{1}{4\pi }\frac{\sigma_{i}}{\sigma_{e}}\int \int \int \vec{\nabla \;’}V_{m}(\vec{r’})\cdot \vec{\nabla \;’}[\frac{1}{\left|\vec{r’}-\vec{r}\right|}]dv,$$

where ∇ ’*V_m_* is the spatial gradient of transmembrane potential, *σ_i_* and *σ_e_* are intracellular and extracellular conductivities, respectively; $\left|\vec{r’}-\vec{r}\right|$ is the distance from the source point (*x*, *y*, *z*) to the measuring point (*x*’, *y*’, *z*’); and *dv* is the volume differential. The EGM signals were obtained at 50 nodes, 28 in the left atrium and 22 in the right atrium (Figure 1e,f), with a temporal resolution of 1 ms. Spectral analysis of the EGM was performed by applying a 40–250 Hz band-pass filter, rectification and low-pass filter at 20 Hz. The Fourier transform was obtained, and dominant frequency (DF) is identified as the highest peak of the power spectrum [60].

## 3. Results

### 3.1. Conduction Velocity and Action Potential Duration

After applying the S1 stimulus under AF conditions without fibrosis and the fourteen fibrosis configurations, the APD_90_ and conduction velocity were measured in the posterior wall of the left atrium. Table 2 summarizes the results for all configurations. The values of fibrosis electrophysiological measures are presented as percentages of the corresponding measures of the model without fibrosis. In the case of AF without fibrosis, the APD_90_ = 118 ms and the estimated conduction velocity was 62 cm/s. For the cases with fibrosis, in general, there was a slight decrease of the APD_90_ and a significant decrease in the conduction velocity. A larger conduction velocity reduction was observed at high fibrosis density, where the configuration with fibrocytes had the largest effect. The reductions in the APD_90_ range from 2% (116 ms) to 5% (112 ms) for the low density, and from 11% (105 ms) to 20% (94 ms) for the high density. No significant variations in the resting potential were observed at low density. A small depolarization (+3 mV) of the resting potential was observed at high density.

For all fibrosis configurations, except the myofibroblast configuration, the conduction velocity decreases as the fibrosis density increases. Such a reduction in the conduction velocity ranges from 21% to 61% for the low density, and from 6% to 82% for the high density. The myofibroblasts configuration presented the lowest reduction in the conduction velocity at low density (i.e., 21%, resulting in 49 cm/s), and at high density (i.e., 6%, resulting in 58 cm/s). On the other hand, the fibrocytes configuration at high density caused the greatest conduction velocity reduction (82%), that corresponds to a conduction velocity of 11 cm/s.

**Figure 2:**
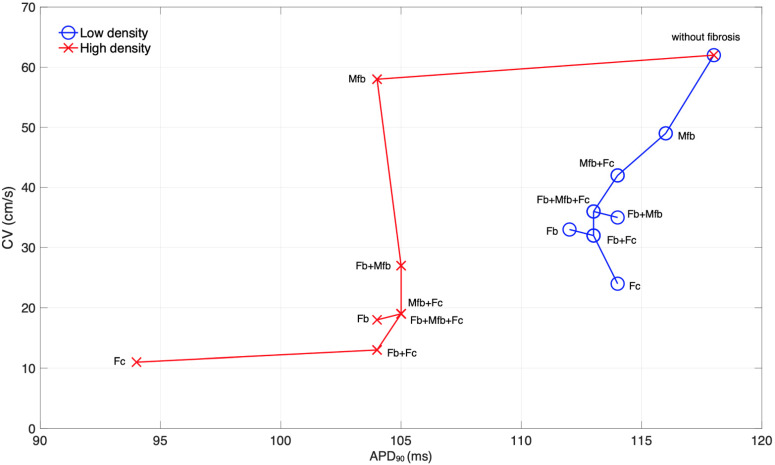
Locus of the conduction velocity (CV) versus APD_90_ for the low (blue color) and high (red color). Each point (APD_90_, CV) represents a fibrosis configuration, including the case without fibrosis. For a given fibrosis density, each point is connected by a line to its closest neighbor. All fibrotic cells configurations are included in the chart with the corresponding label. The notation used is Fb, Mfb and Fc for fibroblasts, myofibroblasts and fibrocytes, respectively.

Figure 2 shows the locus of the conduction velocity (CV) versus APD90 for the low and high fibrosis densities, distinguished by the colors blue and red, respectively. Each pair (APD90, CV) represents a fibrosis configuration, including the case without fibrosis. For a given fibrosis density, each point is connected by a line to its closest neighbor. The “without fibrosis” scenario is considered as the root of the tree. It can be seen in the low and high densities trees, that the progression initiates with myofibroblasts and it advances as other fibrotic cells join the fibrosis configuration. The tree corresponding to the low-density case ends with leaves corresponding to fibroblasts + fibrocytes and fibrocytes configurations. However, the tree corresponding to the high-density case ends with the fibrocytes configuration, suggesting that fibrocytes are relevant during the course of the arrhythmia under both fibrosis densities.

### 3.2. Propagation Dynamics during AF without Fibrosis

The fibrillatory dynamics obtained in the AF model without fibrosis (Video S1) includes two rotors generated in the right atrium. One rotor was located at the junction between the superior vena cava and the Bachmann bundle and dissipated as the episode evolves. The other rotor was observed between the superior vena cava and the interatrial septum, and it migrated towards the top of the terminal crest at the end of the simulation. In the left atrium, plane waves and wavefronts collisions in the posterior wall were observed and, at approximately 3200 ms, a two-rotations re-entry was generated in the base of the appendage.

### 3.3. Propagation Dynamics in the Right Atrium during AF with Fibrosis

For the 14 fibrosis configurations, fibrillatory activity was observed in both atria, where the propagation in the right atrium was similar for all configurations, whereas the left atrium presented distinct dynamics for the different fibrosis configurations. After a few milliseconds of the simulation initiation, all fibrotic scenarios presented rotor activity similar to the case of AF without fibrosis (i.e., a rotor located between the Bachmann bundle and the superior vena cava, and another rotor located between the superior vena cava and the interatrial septum). At the end of the simulations, one of the rotors migrated towards the top of the terminal crest at low fibrosis density in four configurations (fibroblasts, myofibroblasts, fibroblasts + fibrocytes, fibroblasts + myofibroblasts + fibrocytes), and at high fibrosis density in five configurations (fibroblasts, fibrocytes, fibroblasts + myofibroblasts, fibroblasts + fibrocytes, fibroblasts + myofibroblasts + fibrocytes). In the other configurations, only one of the two rotors remained until the end of the simulation. Wavefront collisions in the posterior wall of right atrium were also observed throughout all simulations.

### 3.4. Propagation Dynamics in the Left Atrium during AF with Fibrosis

**Figure 3:**
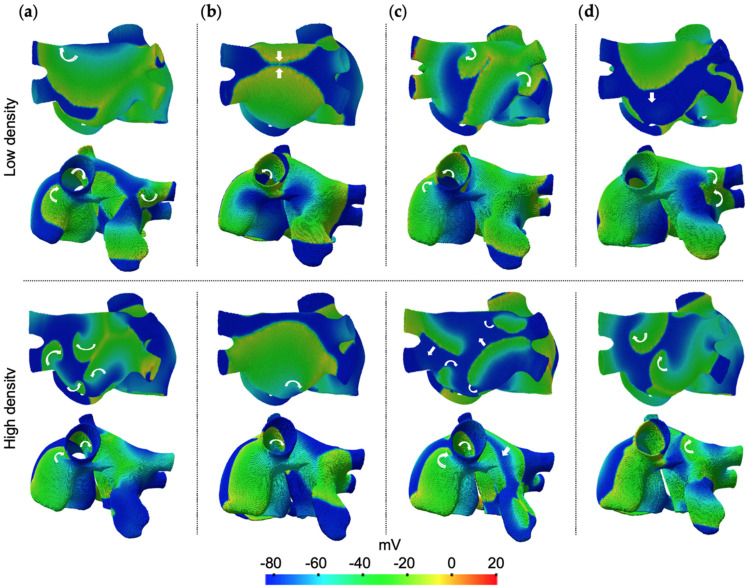
AF simulations with diffuse fibrosis at low (top) and high (bottom) density, for the follow configurations: (**a**) fibroblasts, (**b**) myofibroblasts, (**c**) fibrocytes, (**d**) fibroblasts + myofibroblasts + fibrocytes. White arrows show the direction of waves propagation.

Different propagation patterns in the left atrium were observed according to the fibrosis configuration and density. For the fibroblasts configuration at low density (top of Figure 3a and Video S2), a rotor was generated in the superior wall near to the appendage. At the end of the simulation (at approximately 4000 ms), a figure-of-eight re-entry was observed between the appendage and the base of the left pulmonary veins. At high density (bottom of Figure 3a and Video S3), multiple re-entrant waves (from 2 to 5 waves) collide among each other, leading to chaotic activity mainly located at the posterior and superior walls.

For the myofibroblasts configuration at low density (top of Figure 3b), only plane waves originated in the right atrium and wavefronts collisions in the posterior wall were observed. At high density (bottom of Figure 3b), additional wavefront collisions were sustained and, in the middle of the episode, two transitory re-entries were generated, one in the superior wall and another between the posterior and inferior walls.

For the fibrocytes configuration at low density (top of Figure 3c), the action potential propagation slowed down. Re-entrant activity was generated in the superior wall in the base of the appendage, then (at approximately 2500 ms) two colliding rotors were generated in the posterior wall, one of them evolved into a figure-of-eight re-entry and back into a rotor. At high density (bottom of Figure 3c and Video S4), the reduced velocity conduction yields zigzag propagation in the posterior wall, multiple fragmented wavefronts in the posterior and superior walls, with pivot points in the appendage and anterior and inferior walls.

For the fibroblasts + myofibroblasts configuration at low fibrosis density, a rotor was anchored at the superior wall, in the base of the left pulmonary vein. At approximately 3500 ms, such rotor collided with a migratory rotor generated near the Bachmann bundle, leading to a new rotor sustained in the posterior wall until the end of the simulation. For the high fibrosis density, at approximately 2300 ms, a rotor was observed in the lateral wall near to the left pulmonary veins, and a figure-of-eight re-entry was observed between the posterior and inferior wall until the end of the simulation (not shown in Figure 3).

For the fibroblasts + fibrocytes configuration at low density, at approximately 3000 ms, two rotors were generated. One rotor was observed in the superior wall and migrated to the top of the posterior wall. The other rotor was observed in the base of the appendage. For high fibrosis density, zigzag propagation in the posterior wall was observed until approximately 1200 ms. Subsequently, multiple re-entrant waves (from 3 to 5 waves at time) led to chaotic activity, mainly observed in the posterior and superior walls (not shown in Figure 3).

For the myofibroblasts + fibrocytes configuration at low density, plane waves coming from the right atrium and wavefronts collisions in the posterior and superior wall were observed. For the case of high fibrosis density, a rotor was observed (at approximately 1100 ms) in the superior wall anchored at the base of the left pulmonary vein until the end of the simulation. At approximately 1600 ms, a second rotor was generated in the inferior wall that, subsequently, migrated to the posterior wall (not shown in Figure 3).

For the configuration including the three fibrotic cells (fibroblasts + myofibroblasts + fibrocytes) at low density, a figure-of-eight re-entry was generated between the superior and lateral walls at approximately 3200 ms (top of Figure 3d). For the high fibrosis density, two rotors were observed in the superior wall and in the inferior wall (at approximately 1200 ms). These rotors evolve into multiple re-entrant waves (from 2 to 3 waves) located in the posterior wall at approximately 2000 ms (bottom of Figure 3d and Video S5).

In general, the fibroblasts and fibrocytes favor the generation of re-entrant activity in the left atrium, becoming more chaotic as the density of fibrosis increases. The presence of fibroblasts favors the appearance of multiple rotors and figure-of-eight re-entries. Fibrocytes, by significantly slowing down conduction, favor the zigzag propagation of multiple fragmented and re-entrant wave fronts. On the other hand, myofibroblasts only led to transient re-entrant activity at high density, suggesting that this type of fibrotic cell attenuates the chaotic activity of other fibrotic cells.

### 3.5. Electrograms and DF Analysis

From the AF simulations without fibrosis and with the 14 fibrosis configurations at both densities, 50 EGMs and the corresponding Fourier spectra were calculated. For the case of AF without fibrosis, both atria mostly generated EGMs with single potentials in time, and single and narrow peaks in frequency. In the superior wall of the left atrium and near to superior vena cava, double potentials and fragmentation in the EGM (i.e., two or more potentials with low amplitude, irregular morphology, and cycle length variation) were observed. Such behavior is reflected in the frequency domain with the appearance of multiple spectral peaks. High values of DF were observed in both atria, with a DF mean of 9.2 Hz and zero left-to-right DF gradient.

In the AF simulations with fibrosis, EGMs with single potentials were prevalent. Double potentials and fragmentation in the time domain, with multiple frequency peaks in frequency, were mainly observed near to the Bachmann bundle in the superior wall of the left atrium, at the superior and inferior part of the posterior wall of the left atrium, and near to the superior vena cava in the free wall of the right atrium. Such irregular EMGs can be associated with re-entries, and with breaking and colliding waves, as described in the AF simulations with fibrosis. Additionally, after inspecting the 50 EGMs from the AF episodes at high fibrosis density, we observed a prominent presence of morphological variations, low amplitude, double potentials and fragmented EGMs. For the case of high density of fibrocytes (right side in Figure 3c), a significant lengthening of the cycle length was observed in the EGMs from the left atrium, with a 2:1 conduction between the right atrium and the left atrium and a notorious left-to-right DF gradient.

**Figure 4:**
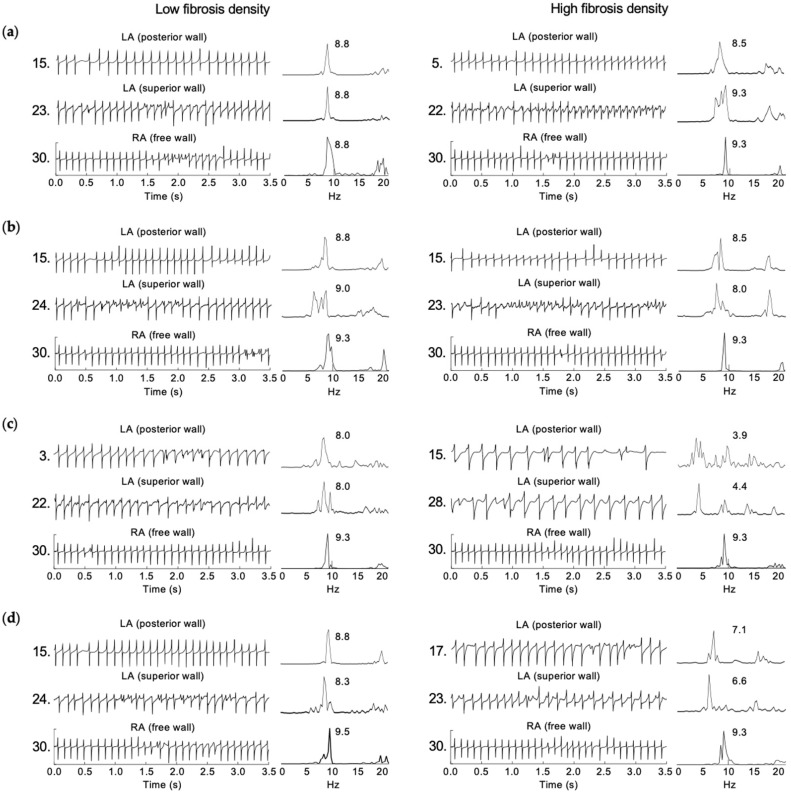
EGMs and their respective spectra at three representative locations in right (RA) and left (LA) atria, for the follow configurations at low (left) and high (right) density: (**a**) fibroblasts, (**b**) myofibroblast, (**c**) fibrocytes, and (**d**) fibroblasts + myofibroblasts + fibrocytes. The DF values are shown.

Figure 4 shows the EGMs and the corresponding spectra at three locations from the right and left atria, for the fibrosis configurations with fibroblasts, myofibroblast, fibrocytes, and fibroblasts + myofibroblasts + fibrocytes, all cases at low (left) and high (right) densities.

For the different fibrosis configurations, the mean values of DF in the right atrium vary between 9.2 Hz and 9.3 Hz, in a similar way as those values obtained for the case of AF without fibrosis. However, in the left atrium, the mean values of DF were reduced for all fibrosis configurations. At low density, the mean values of DF vary between 9.1 Hz for fibroblasts + myofibroblasts to 8.3 Hz for fibrocytes, yielding slight left-to-right DF gradients between 0.2 Hz and 1.0 Hz. At high fibrosis density, the mean values of DF in the left atrium vary between 8.3 Hz for myofibroblasts to 4.8 Hz for fibrocytes, which correspond to left-to-right DF gradients between 1.0 Hz and 4.5 Hz (see Table 3).

**Figure 5:**
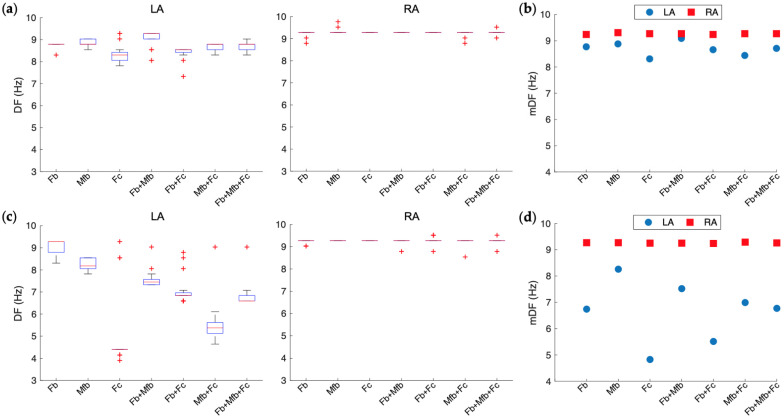
DF boxplots and DF mean (mDF) values in the left (LA) and right (RA) atria, at low (**a**,**b**), and high (**c**,**d**) fibrosis density. All fibrotic cells configurations are included in the chart with the corresponding label. The notation used is Fb, Mfb and Fc for fibroblasts, myofibroblasts and fibrocytes, respectively.

Figure 5 presents the DF boxplots and the DF mean values measured in the left and right atria, at low (Figure 5a,b) and high (Figure 5c,d) fibrosis densities. The boxplots corresponding to the left atrium show scattered values of DF for most fibrosis configurations, with median values between 9.3 Hz (fibroblasts + myofibroblasts) and 8.3 Hz (fibrocytes) at low fibrosis density and between 9.3 Hz (fibroblasts) and 4.4 Hz (fibrocytes) at high density. On the other hand, the boxplots of the right atrium show non-scattered DF values of 9.3 Hz at both densities.

The blue color circles and red color squares in Figure 5b,d, represent the mean values of DF for the left and right atrium, respectively. The left-to-right DF gradient increases with the distance between the red color and blue color marks. The left-to-right DF gradient was larger at high fibrosis density than at low density for all fibrosis configurations. At low density, slight DF gradients could be observed between the right and left atrium, mainly in the fibrocytes (c) and myofibroblasts + fibrocytes (f) configurations (Figure 5b). At high fibrosis density, larger DF gradients were observed, where the highest value was also found for the fibrocytes (c) configuration (Figure 5d).

## 4. Discussion

The findings of our work are as follows: (i) all fibrosis configurations generated a slight APD_90_ shortening and a significant conduction velocity reduction, with a larger effect observed at high fibrosis density. (ii) Increasing fibrosis density from low (6.25%) to high (25%) intensified the vulnerability to multiple re-entries, zigzag propagation, and chaotic activity. (iii) The degree of chaos in the propagation patterns is determined by the fibrosis density: the higher the fibrosis density, the chaotic the propagation. (iv) All fibrosis configurations generated a left-to-right DF gradient between the atria, where small gradients were observed at low densities and significant gradients at high densities. Nevertheless, higher fibrotic density was expected to cause more serious disturbances, given current knowledge in the field, however, the finding that fibrocytes cause more severe conduction disruptions is a novelty. In this line of thought, the main finding of this work is that fibrocytes are the cells with the largest proarrhythmic effect, causing chaotic dynamics with the presence of multiple re-entries and zigzag propagation. Additionally, the fibrocytes configuration also leads to the highest left-to-right DF gradient.

Fibrosis is recognized as an element present in different abnormal disease conditions, such as, diabetes mellitus, obesity, alcoholism, hypertension, obstructive sleep apnea, heart failure, among others [61]. Additionally, the development of fibrosis can be related with other circumstances. Exposure to air pollution contributes to adverse remodeling and worsening myocardial fibrosis [10,11,13,62], degenerative and fibrotic lesions in the myocardium [63]. The myocardial hypertrophy commonly observed in high performance athletes may degenerate in fibrosis remodeling [64]. Furthermore, the severity extent in which fibrosis affects the cardiac function can be submitted to the interaction with multiple factors, such as, genetic conditions [65], gender [66] and ageing [67]. Moreover, in an AF scenario, the presence of fibrosis is expected to increase the proarrhythmic risk [15,18,68,69,70]. Therefore, the studies looking for new insights into the pathophysiological mechanisms of fibrosis yield a wide perspective to the different contexts and conditions in which this structural remodeling occurs. The myocardial perturbation imposed by the fibrosis remodeling involves components at the cellular level. The fibrosis yields the proliferation of fibroblasts and differentiation into myofibroblasts [26,31,34,71]. Additionally, in such disease conditions, the noncardiac-origin fibrocytes migrate to the cardiac tissue and contribute to the fibroblasts population [35]. There is evidence that fibroblasts, myofibroblasts and fibrocytes play a role in the pathophysiology of the arrhythmia [23,24,25,26,27,69,72], where the pathogenesis and sustaining of AF is closely related to the cardiac fibroblast and their myofibroblast differentiation [72,73]. Moreover, increased circulating fibrocytes has served as a biomarker of fibrosis in patients with persistent AF [23,35,74] and the presence of these cells has been correlated with occurrence of conduction blockades [36]. Taking this experimental evidence into account, we assessed the fibroblasts, myofibroblasts and fibrocytes roles on the atrial electrophysiology under persistent AF conditions. The computational approach used for this purpose allows establishing a wide set of fibrosis conditions, in which, the intermingled action of the fibrotic cells was assessed that otherwise would be hard to control and observe in an experimental setup. To the best of our knowledge, there are no reports of computational studies about the effects of fibrocytes, fibroblasts and myofibroblast on the AF propagation patterns. Therefore, our results provide information about the interactions of the fibrotic cells underlying AF fibrosis.

The atrial fibrosis model was implemented using a realistic 3D model of human atria, where the fibrotic cells were coupled to cardiomyocytes and spatially distributed to simulate diffuse fibrosis. The simulated fibrosis density in the left atrium was established based on a clinical study [55], in which 387 patients with AF were classified according to the percentage of fibrosis quantified with magnetic resonance: Utah stage I (<8.5%), Utah stage II (8.6–16%), Utah stage III (16.1–21%), and Utah stage IV (>21.1%). In our simulations, low and high fibrosis densities were considered, corresponding to the Utah fibrosis stages I and IV, respectively [55]. The fibrosis model was implemented only in the left atrium in agreement with clinical observations on the predominance of fibrotic remodeling in the left atrium of AF patients [75] and taking into account that the involvement of left atrial fibrosis in the genesis of AF has been widely reported in animal models and humans [76,77,78,79].

We assessed the effect of the fibrosis configurations on the APD and conduction velocity. Our results suggest that the presence of fibrosis produces a slight APD reduction, in agreement with Trenor et al. [80] and Morgan et al. [81]. A significant reduction in the conduction velocity was observed in all fibrosis configuration, with a larger effect observed at high fibrosis density. The effect of fibroblasts on conduction deceleration have been reported in experimental [31], and simulation studies, where the increase in fibrosis density [81,82] and microfibrosis [83], reduced the conduction velocity. Zhan et al. [84] showed that fibroblasts can alter the conduction velocity with fibrosis densities of 40% and 45%. An in vitro and in silico study [85] showed that an increase of the fibrotic obstacle strand resulted in significant conduction slowing up to 23.6% and a significant increase in the wavefront curvature anisotropy. The relationship between the conduction velocity reduction and the fibrosis density increase was found in most of our configurations, except in the myofibroblasts configuration. This behavior was also observed in [31], with a cell line of myofibroblasts coated on cardiomyocytes strands. On the contrary, studies with primary cultures of ventricular tissue of rats and by simulation [86,87] reported a conduction velocity reduction with increasing myofibroblast density. Further studies should be conducted aiming to elucidate the extent of the effect of myofibroblasts in the atrial electrophysiology.

Since the electrophysiological measures of conduction velocity and APD are biomarkers of the arrhythmic state of the atria, the resulting tree of the locus CV versus APD, provides a view of the progression of such arrhythmic state as the fibrosis configuration changes, by considering the AF without fibrosis scenario as the root of the tree. It is interesting to see that, for the low density, two bifurcations occur. The first bifurcation generates an external branch (fibroblasts + myofibroblasts) which is the last configuration in which the myofibroblasts plays a role in the AF dynamics. The second bifurcation generates two leaves corresponding to fibroblasts and fibrocytes. On the other hand, the high fibrosis tree presents a single bifurcation that generates a leaf corresponding to fibroblasts, and a branch that ends with fibrocytes. It is known that under disease conditions, fibroblasts differentiate into myofibroblasts [30], which agrees with the trees having these cells initiating the fibrosis proarrhythmic effect. Progression continues with myofibroblasts combined with fibrocytes which infiltrate into the atrial tissue as a response of wound healing, followed by intermingled action of the three fibrotic cells. The course of the arrhythmogenicity reaches the more advanced stage when single fibroblasts or fibrocytes are modulating the atrial electrophysiology. It is noteworthy that the tree corresponding to the low-density case, suggest an earlier action of fibrocytes that the one observed for the high-density case. This may be related to the fact that initial states of fibrosis occur at low presence of fibrotic elements of the tissue [55]; at that early stage, circulating fibrocytes are being recruited to contribute to the myofibroblast population [23,35], then, a high fibrosis density occurs at advanced stages. Therefore, our results provide insights into the fibrosis course that is in agreement with experimental and clinical observations [20,31,86,88]. Moreover, the models suggest a relevant role of fibrocytes that should be further investigated.

Our results show fibrosis-relevant effects on electrical propagation patterns during AF. The activity simulated in presence of fibrocytes included zigzag propagation and re-entries. Zigzag activation has been reported in different works. In a high-resolution cardiac mapping study [82], an activation delay was observed between sites perpendicular to the direction of the fiber, leading to a zigzag activation pattern, similarly to that reported by King et al. [20], and Mandapati et al. [89]. The results obtained with the fibroblasts configuration, showing mainly rotors generation, are in agreement with computational studies by McDowell et al. [90], Comtois et al. [91], and Zahid et al. [49], who have shown rotors localized near to areas of fibrosis in 3D atrial models. On the other hand, the activity simulated in the presence of myofibroblasts induced a more regular fibrillatory activity with wavefront collisions. Some studies have reported the role of myofibroblasts in the atrial arrhythmogenesis promoting abnormal impulse conduction [14,31,34].

For each fibrosis configuration, the effect was strengthened by increasing the fibrosis density. The higher the density of fibrosis, the more chaotic was the simulated fibrillatory activity. Several studies suggest that the amount of fibrosis is associated with increased vulnerability to atrial arrhythmias [15,17,18]. In a study with 61 patients [92], higher fibrosis stages were associated with AF recurrence and a poor outcome following ablation therapy. Kheirkhahan et al. [93], in 127 patients with AF underwent ablation, reported a mean fibrosis density in the left atrium of 25.7 ± 9.6%, where the fibrosis formation ≥21% (stage 4 of Utah) was associated with a significantly increased risk of atrial arrhythmia recurrence. In a study where six diffuse fibrosis levels were simulated, based on magnetic resonance imaging, short-term rotors were observed at low density levels, and long-term rotors and multiple re-entries were observed at high-density levels [81]. To our knowledge, no study has taken into account the three types of fibrotic cells that have been experimentally documented.

We also assessed the effect of fibrosis on simulated EGMs. The EGMs recorded during the different fibrosis configurations showed double and fragmented potentials, mainly in regions of the left atrium. This result is in agreement with Jacquemet et al. [83], who showed that myocardial fibrosis affects the EGM morphology, presenting more fragmentation with increasing fibrosis density. Our EGMs analysis showed no left-to-right DF gradient during AF without fibrosis; however, all fibrosis configurations decreased the DF mean in the left atrium at different ratio, leading to left-to-right DF gradients between both atria, which is explained by the fact that fibrosis was implemented only in the left atrium. Small gradients were generated at low fibrosis densities, instead, significant gradients were obtained at high fibrosis densities, the fibrocyte being the fibrotic cell-type with the greatest effect on increasing the DF gradient. Our results are consistent with an experimental study that concluded that diffuse fibrosis and densely fibrotic regions markedly increase the variability and complexity of electrical propagation patterns, leading to DF value reduction [88]. Several studies in both, animals and humans under AF conditions have shown DF gradient between both atria [94,95]. The DF gradients have also been observed in patients with persistent AF with the presence of fibrosis [96,97].

## 5. Limitations

The Courtemanche et al. model was used here to simulate cellular action potentials of human atrial myocytes, which is particularly suitable for the human left atrium, the limitations of this model have been discussed elsewhere [98]. Our results were obtained using a virtual atrial model with detailed anatomical structure and physiology (i.e., electrophysiology, anatomy, fiber direction, anisotropy and heterogeneity). However, it is a general atrial model. Future studies should incorporate patient-specific atrial models since the variability between patients is a common clinical problem when diagnosing atrial arrhythmias. Similarly, the simulated fibrosis configurations do not correspond to a reconstruction from patient-specific images. Despite the incorporation of the electrical and structural remodeling observed in patients with persistent AF through different configurations of diffuse fibrosis, including the three types of fibrotic cells, the patchy and compact fibrosis were not considered. Future investigations should assess the three different fibrosis textures and fibrosis accounting for the four Utah stages, under distinct percentage ranges for each stage. Finally, the fibrocyte cell was represented using a fibroblast model with very low conductivity, based on experimental evidence of conduction blocks in atrial tissue with the presence of fibrocytes. Future studies should consider a specific electrophysiological model for the fibrocyte.

## 6. Conclusions

The effect of the configuration and density of three types of fibrotic cells on the fibrillatory dynamics during persistent AF was evaluated by implementing a realistic 3D model of human atria. A significant conduction velocity reduction at high fibrosis density increased vulnerability to multiple re-entries, zigzag propagation, and chaotic activity in fibrillatory dynamics. The chaotic propagation patterns and the left-to-right DF gradients depend on the configuration and density of fibrosis, where a larger proarrhythmic effect was observed at high fibrosis densities in the presence of fibrocytes. Our results contribute to a better understanding of the roles of diffuse fibrosis density and the type and configuration of fibrotic cells on fibrillatory dynamics that could help in the development of newer and more effective treatment approaches for persistent AF.

## Figures and Tables

**Table 1:** Membrane capacitance and conductivity assigned to myocytes and fibrotic cells.

Cell	Capacitance	Conductivity	References
Cardiomyocyte	100 pF	0.26 S/cm	[38,51]
Fibroblast	6.3 pF	0.078 S/cm	[45,46,47,49]
Myofibroblast	50.4 pF	0.078 S/cm	[46,49]
Fibrocyte	6.3 pF	0.024 S/cm	[36,46,47,50,52]

**Table 2:** Fibrosis effects on the APD_90_ and conduction velocity (CV), for each fibrosis configuration at low and high density.

Fibrosis Configuration	APD_90_ (ms)		APD_90_ Reduction		CV (cm/s)		CV Reduction	
	Low Density	High Density	Low Density	High Density	Low Density	High Density	Low Density	High Density
Without fibrosis	118		-		62		-	
Fibroblasts	112	104	5%	12%	33	18	47%	71%
Myofibroblasts	116	104	2%	12%	49	58	21%	6%
Fibrocytes	114	94	3%	20%	24	11	61%	82%
Fibroblasts + myofibroblasts	114	105	3%	11%	35	27	44%	56%
Fibroblasts + fibrocytes	113	104	4%	12%	32	13	49%	79%
Myofibroblasts + fibrocytes	114	105	3%	11%	42	19	32%	70%
Fibroblasts + myofibroblasts + fibrocytes	113	105	4%	11%	36	19	42%	70%

**Table 3:** Values of mDF and left-to-right DF gradient, at low and high density with different fibrosis configuration.

Fibrosis Configuration	Fibrosis Density	LA mDF (Hz)	RA mDF (Hz)	Right-to-Left DF Gradient (Hz)
Without fibrosis	-	9.2	9.2	0.0
Fibroblasts	Low	8.8	9.2	0.4
	High	6.7	9.3	2.6
Myofibroblasts	Low	8.9	9.3	0.4
	High	8.3	9.3	1.0
Fibrocytes	Low	8.3	9.3	1.0
	High	4.8	9.3	4.5
Fibroblasts + myofibroblasts	Low	9.1	9.3	0.2
	High	7.5	9.3	1.8
Fibrocytes + fibroblasts	Low	8.7	9.2	0.5
	High	5.5	9.2	3.7
Fibrocytes + myofibroblasts	Low	8.4	9.3	0.9
	High	7.0	9.3	2.3
Fibrocytes + myofibroblasts + fibrocytes	Low	8.7	9.3	0.6
	High	6.8	9.3	2.5

## Data Availability

All data generated in this study are depicted as individual values within the figures. Original data are available on reasonable request from the corresponding authors.

## Supplementary Materials

The following are available online at https://www.mdpi.com/article/10.3390/cells10102769/s1. Video S1: atrial fibrillation without fibrosis; Video S2: fibroblasts configuration at low density; Video S3: fibroblasts configuration at high density, Video S4: fibrocytes configuration at high density, Video S5: fibroblasts + myofibroblasts + fibrocytes at high density.

## References

[1] Nattel S., Harada M. (2014). Atrial remodeling and atrial fibrillation: Recent advances and translational perspectives. J. Am. Coll. Cardiol..

[2] Stewart S., Murphy N., Walker A., McGuire A., McMurray J.J.V. (2004). Cost of an emerging epidemic: An economic analysis of atrial fibrillation in the UK. Heart.

[3] Andrade J., Khairy P., Dobrev D., Nattel S. (2014). The Clinical Profile and Pathophysiology of Atrial Fibrillation. Circ. Res..

[4] Haissaguerre M., Jais P., Shah D.C., Takahashi A., Hocini M., Quiniou G., Garrigue S., Le Mouroux A., Le Métayer P., Clémenty J. (1998). Spontaneous Initiation of Atrial Fibrillation by Ectopic Beats Originating in the Pulmonary Veins. N. Engl. J. Med..

[5] Moe G.K., Rheinboldt W.C., Abildskov J. (1964). A computer model of atrial fibrillation. Am. Heart J..

[6] Jalife J. (2002). Mother rotors and fibrillatory conduction: A mechanism of atrial fibrillation. Cardiovasc. Res..

[7] Wijffels M.C., Kirchhof C.J., Dorland R., Allessie M.A. (1995). Atrial fibrillation begets atrial fibrillation. A study in awake chronically instrumented goats. Circulation.

[8] Allessie M., Ausma J., Schotten U. (2002). Electrical, contractile and structural remodeling during atrial fibrillation. Cardiovasc. Res..

[9] Jalife J., Kaur K. (2015). Atrial remodeling, fibrosis, and atrial fibrillation. Trends Cardiovasc. Med..

[10] Newby D.E., Mannucci P.M., Tell G.S., Baccarelli A.A., Brook R.D., Donaldson K., Forastiere F., Franchini M., Franco O.H., Graham I. (2015). Expert position paper on air pollution and cardiovascular disease. Eur. Heart J..

[11] Liu Y., Goodson J.M., Zhang B., Chin M.T. (2015). Air pollution and adverse cardiac remodeling: Clinical effects and basic mechanisms. Front. Physiol..

[12] Brook R.D., Rajagopalan S., Pope C.A., Brook J.R., Bhatnagar A., Diez-Roux A.V., Holguin F., Hong Y., Luepker R.V., Mittleman M.A. (2010). Particulate Matter Air Pollution and Cardiovascular Disease. Circulation.

[13] de Oliveira-Fonoff A.M., Mady C., Pessoa F.G., Fonseca K.C.B., Salemi V.M.C., Fernandes F., Saldiva P.H.N., Ramires F.J.A. (2017). The role of air pollution in myocardial remodeling. PLoS ONE.

[14] Rohr S. (2009). Myofibroblasts in diseased hearts: New players in cardiac arrhythmias?. Hear. Rhythm.

[15] Xu J., Cui G., Esmailian F., Plunkett M., Marelli D., Ardehali A., Odim J., Laks H., Sen L. (2004). Atrial Extracellular Matrix Remodeling and the Maintenance of Atrial Fibrillation. Circulation.

[16] Fukumoto K., Habibi M., Ipek E.G., Zahid S., Khurram I.M., Zimmerman S.L., Zipunnikov V., Spragg D., Ashikaga H., Trayanova N. (2016). Association of Left Atrial Local Conduction Velocity with Late Gadolinium Enhancement on Cardiac Magnetic Resonance in Patients with Atrial Fibrillation. Circ. Arrhythm. Electrophysiol..

[17] Burstein B., Comtois P., Michael G., Nishida K., Villeneuve L., Yeh Y.-H., Nattel S. (2009). Changes in Connexin Expression and the Atrial Fibrillation Substrate in Congestive Heart Failure. Circ. Res..

[18] Akoum N., Daccarett M., McGann C., Segerson N., Vergara G., Kuppahally S., Badger T., Burgon N., Haslam T., Kholmovski E. (2011). Atrial fibrosis helps select the appropriate patient and strategy in catheter ablation of atrial fibrillation: A DE-MRI guided approach. J. Cardiovasc. Electrophysiol..

[19] de Jong S., van Veen T.A.B., van Rijen H.V.M., de Bakker J.M.T. (2011). Fibrosis and Cardiac Arrhythmias. J. Cardiovasc. Pharmacol..

[20] de Bakker J.M., van Capelle F.J., Janse M.J., Tasseron S., Vermeulen J.T., de Jonge N., Lahpor J.R. (1993). Slow conduction in the infarcted human heart. “Zigzag” course of activation. Circulation.

[21] Kawara T., Derksen R., de Groot J.R., Coronel R., Tasseron S., Linnenbank A.C., Hauer R.N.W., Kirkels H., Janse M.J., de Bakker J.M.T. (2001). Activation Delay after Premature Stimulation in Chronically Diseased Human Myocardium Relates to the Architecture of Interstitial Fibrosis. Circulation.

[22] Ten Tusscher K.H.W.J., Panfilov A.V. (2007). Influence of diffuse fibrosis on wave propagation in human ventricular tissue. EP Eur..

[23] Xie X., Liu Y., Gao S., Wu B., Hu X., Chen J. (2014). Possible Involvement of Fibrocytes in Atrial Fibrosis in Patients With Chronic Atrial Fibrillation. Circ. J..

[24] Rücker-Martin C. (2002). Dedifferentiation of atrial myocytes during atrial fibrillation: Role of fibroblast proliferation in vitro. Cardiovasc. Res..

[25] Camelliti P., Green C.R., LeGrice I., Kohl P. (2004). Fibroblast network in rabbit sinoatrial node: Structural and functional identification of homogeneous and heterogeneous cell coupling. Circ. Res..

[26] Camelliti P., Borg T., Kohl P. (2005). Structural and functional characterisation of cardiac fibroblasts. Cardiovasc. Res..

[27] Chilton L., Giles W.R., Smith G.L. (2007). Evidence of intercellular coupling between co-cultured adult rabbit ventricular myocytes and myofibroblasts. J. Physiol..

[28] Aujla P.K., Kassiri Z. (2021). Diverse origins and activation of fibroblasts in cardiac fibrosis. Cell. Signal..

[29] Yue L., Xie J., Nattel S. (2011). Molecular determinants of cardiac fibroblast electrical function and therapeutic implications for atrial fibrillation. Cardiovasc. Res..

[30] Cabanas-grandío P., Bisbal F. (2015). Current Role and Future Prospects of Magnetic Resonance Imaging in the Field of Atrial Fibrillation Ablation. J. Atr. Fibrillation.

[31] Miragoli M., Gaudesius G., Rohr S. (2006). Electrotonic Modulation of Cardiac Impulse Conduction by Myofibroblasts. Circ. Res..

[32] Miragoli M., Glukhov A.V. (2015). Atrial Fibrillation and Fibrosis: Beyond the Cardiomyocyte Centric View. BioMed Res. Int..

[33] Krul S.P.J., Berger W.R., Smit N.W., van Amersfoorth S.C.M., Driessen A.H.G., van Boven W.J., Fiolet J.W.T., van Ginneken A.C.G., van der Wal A.C., de Bakker J.M.T. (2015). Atrial Fibrosis and Conduction Slowing in the Left Atrial Appendage of Patients Undergoing Thoracoscopic Surgical Pulmonary Vein Isolation for Atrial Fibrillation. Circ. Arrhythm. Electrophysiol..

[34] Miragoli M., Salvarani N., Rohr S. (2007). Myofibroblasts Induce Ectopic Activity in Cardiac Tissue. Circ. Res..

[35] Mori L., Bellini A., Stacey M.A., Schmidt M., Mattoli S. (2005). Fibrocytes contribute to the myofibroblast population in wounded skin and originate from the bone marrow. Exp. Cell Res..

[36] Lin R.-J., Su Z.-Z., Liang S.-M., Chen Y.-Y., Shu X.-R., Nie R.-Q., Wang J.-F., Xie S.-L. (2016). Role of Circulating Fibrocytes in Cardiac Fibrosis. Chin. Med. J..

[37] Jalife J. (2014). Mechanisms of persistent atrial fibrillation. Curr. Opin. Cardiol..

[38] Courtemanche M., Ramirez R.J., Nattel S. (1998). Ionic mechanisms underlying human atrial action potential properties: Insights from a mathematical model. Am. J. Physiol.-Heart Circ. Physiol..

[39] Kneller J., Zou R., Vigmond E.J., Wang Z., Leon L.J., Nattel S. (2002). Cholinergic Atrial Fibrillation in a Computer Model of a Two-Dimensional Sheet of Canine Atrial Cells With Realistic Ionic Properties. Circ. Res..

[40] Caballero R., de la Fuente M.G., Gómez R., Barana A., Amorós I., Dolz-Gaitón P., Osuna L., Almendral J., Atienza F., Fernández-Avilés F. (2010). In Humans, Chronic Atrial Fibrillation Decreases the Transient Outward Current and Ultrarapid Component of the Delayed Rectifier Current Differentially on Each Atria and Increases the Slow Component of the Delayed Rectifier Current in Both. J. Am. Coll. Cardiol..

[41] Bosch R.F., Zeng X., Grammer J.B., Popovic K., Mewis C., Kühlkamp V. (1999). Ionic mechanisms of electrical remodeling in human atrial fibrillation. Cardiovasc. Res..

[42] Van Wagoner D.R., Pond A.L., Lamorgese M., Rossie S.S., McCarthy P.M., Nerbonne J.M. (1999). Atrial L-type Ca2+ currents and human atrial fibrillation. Circ. Res..

[43] Colman M.A., Aslanidi O.V., Kharche S., Boyett M.R., Garratt C., Hancox J.C., Zhang H. (2013). Pro-arrhythmogenic effects of atrial fibrillation-induced electrical remodelling: Insights from the three-dimensional virtual human atria. J. Physiol..

[44] Martinez-Mateu L., Romero L., Ferrer-Albero A., Sebastian R., Rodríguez Matas J.F., Jalife J., Berenfeld O., Saiz J. (2018). Factors affecting basket catheter detection of real and phantom rotors in the atria: A computational study. PLoS Comput. Biol..

[45] Andrew MacCannell K., Bazzazi H., Chilton L., Shibukawa Y., Clark R.B., Giles W.R. (2007). A Mathematical Model of Electrotonic Interactions between Ventricular Myocytes and Fibroblasts. Biophys. J..

[46] Ashihara T., Haraguchi R., Nakazawa K., Namba T., Ikeda T., Nakazawa Y., Ozawa T., Ito M., Horie M., Trayanova N.A. (2012). The role of fibroblasts in complex fractionated electrograms during persistent/permanent atrial fibrillation: Implications for electrogram-based catheter ablation. Circ. Res..

[47] Maleckar M.M., Greenstein J.L., Giles W.R., Trayanova N.A. (2009). Electrotonic coupling between human atrial myocytes and fibroblasts alters myocyte excitability and repolarization. Biophys. J..

[48] Chilton L., Ohya S., Freed D., George E., Drobic V., Shibukawa Y., MacCannell K.A., Imaizumi Y., Clark R.B., Dixon I.M.C. (2005). K^+^ currents regulate the resting membrane potential, proliferation, and contractile responses in ventricular fibroblasts and myofibroblasts. Am. J. Physiol. Circ. Physiol..

[49] Zahid S., Cochet H., Boyle P.M., Schwarz E.L., Whyte K.N., Vigmond E.J., Dubois R., Hocini M., Haïssaguerre M., Jaïs P. (2016). Patient-derived models link re-entrant driver localization in atrial fibrillation to fibrosis spatial pattern. Cardiovasc. Res..

[50] Sung R.J., Lauer M.R. (2000). Fundamental Approaches to the Management of Cardiac Arrhythmias. Fundamental Approaches to the Management of Cardiac Arrhythmias.

[51] van der Velden H., Ausma J., Rook M.B., Hellemons A.J.C.G.M., van Veen T.A.A.B., Allessie M.A., Jongsma H.J. (2000). Gap junctional remodeling in relation to stabilization of atrial fibrillation in the goat. Cardiovasc. Res..

[52] Bucala R., Spiegel L.A., Chesney J., Hogan M., Cerami A. (1994). Circulating fibrocytes define a new leukocyte subpopulation that mediates tissue repair. Mol. Med..

[53] Tobón C., Ruiz-Villa C., Heidenreich E., Romero L., Hornero F., Saiz J. (2013). A Three-Dimensional Human Atrial Model with Fiber Orientation. Electrograms and Arrhythmic Activation Patterns Relationship. PLoS ONE.

[54] Ferrer A., Sebastián R., Sánchez-Quintana D., Rodríguez J.F., Godoy E.J., Martínez L., Saiz J. (2015). Detailed Anatomical and Electrophysiological Models of Human Atria and Torso for the Simulation of Atrial Activation. PLoS ONE.

[55] Daccarett M., Badger T.J., Akoum N., Burgon N.S., Mahnkopf C., Vergara G., Kholmovski E., McGann C.J., Parker D., Brachmann J. (2011). Association of left atrial fibrosis detected by delayed-enhancement magnetic resonance imaging and the risk of stroke in patients with atrial fibrillation. J. Am. Coll. Cardiol..

[56] Gomez J.F., Cardona K., Martinez L., Saiz J., Trenor B. (2014). Electrophysiological and Structural Remodeling in Heart Failure Modulate Arrhythmogenesis. 2D Simulation Study. PLoS ONE.

[57] Henriquez C.S., Papazoglou A.A. (1996). Using computer models to understand the roles of tissue structure and membrane dynamics in arrhythmogenesis. Proc. IEEE.

[58] Heidenreich E.A., Ferrero J.M., Doblaré M., Rodríguez J.F. (2010). Adaptive macro finite elements for the numerical solution of monodomain equations in cardiac electrophysiology. Ann. Biomed. Eng..

[59] Ferrero J.M., Ferrero J.M.J., Saiz J., Arnau A. (1994). Bioelectrónica. Señales Bioeléctricas.

[60] Everett T.H., Kok L.C., Vaughn R.H., Moorman J.R., Haines D.E. (2001). Frequency domain algorithm for quantifying atrial fibrillation organization to increase defibrillation efficacy. IEEE Trans. Biomed. Eng..

[61] Lau D.H., Linz D., Sanders P. (2019). New Findings in Atrial Fibrillation Mechanisms. Card. Electrophysiol. Clin..

[62] Chen T.-L., Liao J.-W., Chan W.-H., Hsu C.-Y., Yang J.-D., Ueng T.-H. (2013). Induction of cardiac fibrosis and transforming growth factor-β1 by motorcycle exhaust in rats. Inhal. Toxicol..

[63] Wold L.E., Ying Z., Hutchinson K.R., Velten M., Gorr M.W., Velten C., Youtz D.J., Wang A., Lucchesi P.A., Sun Q. (2012). Cardiovascular Remodeling in Response to Long-Term Exposure to Fine Particulate Matter Air Pollution. Circ. Heart Fail..

[64] Sharykin A.S., Badtieva V.A., Trunina I.I., Osmanov I.M. (2019). Myocardial fibrosis—A new component of heart remodeling in athletes?. Cardiovasc. Ther. Prev..

[65] Doñate Puertas R., Millat G., Ernens I., Gache V., Chauveau S., Morel E., Christin E., Couturier N., Devaux Y., Chevalier P. (2018). Atrial Structural Remodeling Gene Variants in Patients with Atrial Fibrillation. BioMed Res. Int..

[66] Tian X.-T., Xu Y.-J., Yang Y.-Q. (2020). Gender Differences in Arrhythmias: Focused on Atrial Fibrillation. J. Cardiovasc. Transl. Res..

[67] Cochet H., Mouries A., Nivet H., Sacher F., Derval N., Denis A., Merle M., Relan J., Hocini M., Haïssaguerre M. (2015). Age, Atrial Fibrillation, and Structural Heart Disease Are the Main Determinants of Left Atrial Fibrosis Detected by Delayed-Enhanced Magnetic Resonance Imaging in a General Cardiology Population. J. Cardiovasc. Electrophysiol..

[68] Ma J., Chen Q., Ma S. (2021). Left atrial fibrosis in atrial fibrillation: Mechanisms, clinical evaluation and management. J. Cell. Mol. Med..

[69] Li S., Gao Y., Liu Y., Li J., Yang X., Hu R., Liu J., Zhang Y., Zuo K., Li K. (2020). Myofibroblast-Derived Exosomes Contribute to Development of a Susceptible Substrate for Atrial Fibrillation. Cardiology.

[70] Lüscher T.F. (2020). Challenges in atrial fibrillation: Detection, alert systems, fibrosis, and infection. Eur. Heart J..

[71] Kohl P., Camelliti P., Burton F.L., Smith G.L. (2005). Electrical coupling of fibroblasts and myocytes: Relevance for cardiac propagation. J. Electrocardiol..

[72] Poulet C., Künzel S., Büttner E., Lindner D., Westermann D., Ravens U. (2016). Altered physiological functions and ion currents in atrial fibroblasts from patients with chronic atrial fibrillation. Physiol. Rep..

[73] He L., Liu R., Yue H., Ren S., Zhu G., Guo Y., Qin C. (2021). Actin-granule formation is an additional step in cardiac myofibroblast differentiation. Ann. Transl. Med..

[74] Liu Y., Niu X., Yin X., Liu Y., Han C., Yang J., Huang X., Yu X., Gao L., Yang Y. (2018). Elevated Circulating Fibrocytes Is a Marker of Left Atrial Fibrosis and Recurrence of Persistent Atrial Fibrillation. J. Am. Heart Assoc..

[75] Corradi D., Callegari S., Benussi S., Maestri R., Pastori P., Nascimbene S., Bosio S., Dorigo E., Grassani C., Rusconi R. (2005). Myocyte changes and their left atrial distribution in patients with chronic atrial fibrillation related to mitral valve disease. Hum. Pathol..

[76] Xiao H.D., Fuchs S., Campbell D.J., Lewis W., Dudley S.C., Kasi V.S., Hoit B.D., Keshelava G., Zhao H., Capecchi M.R. (2004). Mice with cardiac-restricted angiotensin-converting enzyme (ACE) have atrial enlargement, cardiac arrhythmia, and sudden death. Am. J. Pathol..

[77] Anyukhovsky E., Sosunov E.A., Plotnikov A., Gainullin R.Z., Jhang J.S., Marboe C.C., Rosen M.R. (2002). Cellular electrophysiologic properties of old canine atria provide a substrate for arrhythmogenesis. Cardiovasc. Res..

[78] Frustaci A., Chimenti C., Bellocci F., Morgante E., Russo M.A., Maseri A. (1997). Histological substrate of atrial biopsies in patients with lone atrial fibrillation. Circulation.

[79] Boldt A., Wetzel U., Lauschke J., Weigl J., Gummert J., Hindricks G., Kottkamp H., Dhein S. (2004). Fibrosis in left atrial tissue of patients with atrial fibrillation with and without underlying mitral valve disease. Heart.

[80] Mora M.T., Ferrero J.M., Gomez J.F., Sobie E.A., Trenor B. (2018). Ca^2+^ Cycling Impairment in Heart Failure Is Exacerbated by Fibrosis: Insights Gained From Mechanistic Simulations. Front. Physiol..

[81] Morgan R., Colman M.A., Chubb H., Seemann G., Aslanidi O.V. (2016). Slow Conduction in the Border Zones of Patchy Fibrosis Stabilizes the Drivers for Atrial Fibrillation: Insights from Multi-Scale Human Atrial Modeling. Front. Physiol..

[82] de Bakker J.M.T., van Rijen H.M.V. (2006). Continuous and Discontinuous Propagation in Heart Muscle. J. Cardiovasc. Electrophysiol..

[83] Jacquemet V., Henriquez C.S. (2009). Genesis of complex fractionated atrial electrograms in zones of slow conduction: A computer model of microfibrosis. Hear. Rhythm.

[84] Zhan H., Xia L., Shou G., Zang Y., Liu F., Crozier S. (2014). Fibroblast proliferation alters cardiac excitation conduction and contraction: A computational study. J. Zhejiang Univ. Sci. B.

[85] Gokhale T.A., Asfour H., Verma S., Bursac N., Henriquez C.S. (2018). Microheterogeneity-induced conduction slowing and wavefront collisions govern macroscopic conduction behavior: A computational and experimental study. PLoS Comput. Biol..

[86] Jousset F., Maguy A., Rohr S., Kucera J.P. (2016). Myofibroblasts electrotonically coupled to cardiomyocytes alter conduction: Insights at the cellular level from a detailed in silico tissue structure model. Front. Physiol..

[87] Zlochiver S., Muñ V., Vikstrom K.L., Taffet S.M., Berenfeld O., Jalife J. (2008). Electrotonic Myofibroblast-to-Myocyte Coupling Increases Propensity to Reentrant Arrhythmias in Two-Dimensional Cardiac Monolayers. Biophys. J..

[88] Tanaka K., Zlochiver S., Vikstrom K.L., Yamazaki M., Moreno J., Klos M., Zaitsev A.V., Vaidyanathan R., Auerbach D.S., Landas S. (2007). Spatial Distribution of Fibrosis Governs Fibrillation Wave Dynamics in the Posterior Left Atrium During Heart Failure. Circ. Res..

[89] King J., Huang C., Frase J. (2013). Determinants of myocardial conduction velocity: Implications for arrhythmogenesis. Front. Physiol..

[90] McDowell K.S., Zahid S., Vadakkumpadan F., Blauer J., MacLeod R.S., Trayanova N.A. (2015). Virtual electrophysiological study of atrial fibrillation in fibrotic remodeling. PLoS ONE.

[91] Comtois P., Nattel S. Interactions between cardiac fibrosis spatial pattern and ionic remodeling on electrical wave propagation. Proceedings of the 2011 Annual International Conference of the IEEE Engineering in Medicine and Biology Society.

[92] Luetkens J.A., Wolpers A.C., Beiert T., Kuetting D., Dabir D., Homsi R., Meendermann H., Dayé N.A., Knappe V., Karsdal M. (2018). Cardiac magnetic resonance using late gadolinium enhancement and atrial T1 mapping predicts poor outcome in patients with atrial fibrillation after catheter ablation therapy. Sci. Rep..

[93] Kheirkhahan M., Baher A., Goldooz M., Kholmovski E.G., Morris A.K., Csecs I., Chelu M.G., Wilson B.D., Marrouche N.F. (2020). Left atrial fibrosis progression detected by LGE-MRI after ablation of atrial fibrillation. Pacing Clin. Electrophysiol..

[94] Mansour M., Mandapati R., Berenfeld O., Chen J., Samie F.H., Jalife J. (2001). Left-to-right gradient of atrial frequencies during acute atrial fibrillation in the isolated sheep heart. Circulation.

[95] Filgueiras-Rama D., Price N.F., Martins R.P., Yamazaki M., Avula U.M.R., Kaur K., Kalifa J., Ennis S., Hwang E., Devabhaktuni V. (2012). Long-Term Frequency Gradients During Persistent Atrial Fibrillation in Sheep Are Associated With Stable Sources in the Left Atrium. Circ. Arrhythm. Electrophysiol..

[96] Traykov V.B., Pap R., Saghy L. (2012). Frequency domain mapping of atrial fibrillation-methodology, experimental data and clinical implications. Curr. Cardiol. Rev..

[97] Berenfeld O. (2010). Ionic and substrate mechanism of atrial fibrillation: Rotors and the exitación frequency approach. Arch. Cardiol. Mex..

[98] Wilhelms M., Hettmann H., Maleckar M.M., Koivumäki J.T., Dössel O., Seemann G. (2012). Benchmarking electrophysiological models of human atrial myocytes. Front. Physiol..

